# Predictive value of human chorionic gonadotrophin in the outcome of early pregnancy achieved by assisted reproductive technology: A summative assessment

**DOI:** 10.4103/0974-1208.74165

**Published:** 2010

**Authors:** Viroj Wiwanitkit

**Affiliations:** Wiwanitkit House, Bangkhae, Bangkok - 10160, Thailand

Sir,

Prediction of the early pregnancy achieved by assisted reproductive technology is an important practice in the field. There are several ways to predict. Using of blood tests is an important approach. The use of human chorionic gonadotrophin (hCG) level determination is a test of interest. There are some previous reports on using this specific test for this prediction purpose. Here, the author performed a summative analysis to assess the value of human chorionic gonadotrophin (hCG). The author performed a retrospective literature search on this specific topic to find published papers in PubMed and Scopus. The derived 3 publications on 810 cases are extracted for data on diagnostic properties and summative assessment was done.[1–3] Indeed, there are two other publications on this area that are excluded. One is due to the different period of hCG measurement from other published papers (on the second week)[4] and the other is due to lack of English text.[5] Based on this assessment, the summative sensitivity and specificity for prediction of viable pregnancy are equal to 85.7 and 72.4%, respectively. For differentiation between single and multiple pregnancies in viable group, the summative sensitivity and specificity for prediction of viable pregnancy are equal to 84.3 and 79.2%. Based on this appraisal, it can be seen that hCG measurement gives only fair diagnostic property for determining the outcome of early pregnancy achieved by assisted reproductive technology.
